# Varying negative work assistance at the ankle with a soft exosuit during loaded walking

**DOI:** 10.1186/s12984-017-0267-5

**Published:** 2017-06-26

**Authors:** Philippe Malcolm, Sangjun Lee, Simona Crea, Christopher Siviy, Fabricio Saucedo, Ignacio Galiana, Fausto A. Panizzolo, Kenneth G. Holt, Conor J. Walsh

**Affiliations:** 1 John A. Paulson School of Engineering and Applied Sciences, Harvard University, 29 Oxford Street, Cambridge, MA 02138 USA; 2 Wyss Institute for Biologically Inspired Engineering at Harvard, 3 Blackfan Circle, Boston, MA 02115 USA; 3 Department of Biomechanics and Center for Research in Human Movement Variability, University of Nebraska Omaha, Omaha, NE 68182 USA; 4 The BioRobotics Institute, Scuola Superiore Sant’Anna, viale Rinaldo Piaggio, Pontedera (PI), Italy; 5 Sargent College of Health and Rehabilitation Science, Boston University, Boston, MA 02215 USA

## Abstract

**Background:**

Only very recently, studies have shown that it is possible to reduce the metabolic rate of unloaded and loaded walking using robotic ankle exoskeletons. Some studies obtained this result by means of high positive work assistance while others combined negative and positive work assistance. There is no consensus about the isolated contribution of negative work assistance. Therefore, the aim of the present study is to examine the effect of varying negative work assistance at the ankle joint while maintaining a fixed level of positive work assistance with a multi-articular soft exosuit.

**Methods:**

We tested eight participants during walking at 1.5 ms^−1^ with a 23-kg backpack. Participants wore a version of the exosuit that assisted plantarflexion via Bowden cables tethered to an off-board actuation platform. In four active conditions we provided different rates of exosuit bilateral ankle negative work assistance ranging from 0.015 to 0.037 W kg^−1^ and a fixed rate of positive work assistance of 0.19 W kg^−1^.

**Results:**

All active conditions significantly reduced metabolic rate by 11 to 15% compared to a reference condition, where the participants wore the exosuit but no assistance was provided. We found no significant effect of negative work assistance. However, there was a trend (*p* = .08) toward greater reduction in metabolic rate with increasing negative work assistance, which could be explained by observed reductions in biological ankle and hip joint power and moment.

**Conclusions:**

The non-significant trend of increasing negative work assistance with increasing reductions in metabolic rate motivates the value in further studies on the relative effects of negative and positive work assistance. There may be benefit in varying negative work over a greater range or in isolation from positive work assistance.

**Electronic supplementary material:**

The online version of this article (doi:10.1186/s12984-017-0267-5) contains supplementary material, which is available to authorized users.

## Background

Through regular training, individuals in certain specialized professions are able to walk fast with heavy loads. For example, soldiers are able to walk with loads ranging from 30 to 100% their body weight [1]. Nevertheless, overloading dramatically increases the metabolic rate of walking [2] which in turn causes earlier onset of fatigue and increased injury risk [3].

Different labs have developed robotic exoskeletons intended to unburden individuals carrying load. While early studies with rigid full-leg exoskeletons demonstrated the ability to unload a backpack load from a wearer [4], these systems caused an increase in metabolic effort compared to normal walking [5, 6]. However, over the past years, a number of labs successfully developed early prototype devices that achieved reductions in metabolic rate of unloaded [7–9] and loaded [10, 11] walking, and increase in maximal load-carrying performance [12] in controlled treadmill experiments. One explanation as to why reductions in metabolic rate were achieved only recently could be that earlier exoskeletons were too heavy [10, 13], with rigid structures over the length of the whole leg that caused wearers to deviate significantly from their natural gait. Recent single joint designs [7, 8, 10, 14–17] weigh less and provide targeted assistance at the wearer’s joints, rather than transferring load directly to the ground. Apart from the improved designs, an additional factor that contributed to the recent successes of these assistive devices was knowledge gained from parameter sweep studies that explored the effects of different actuation parameters in a systematic way [7, 8, 18–20].

Two devices that produced metabolic rate reduction versus normal walking provided high positive work assistance at the ankle joint [7, 9, 10]. Another device from Collins et al., however, produced metabolic reduction by means of an intermediate level of moment assistance during both the period of negative and positive ankle work using an unpowered ankle exoskeleton with a clutchable spring [8].

Following the path of wearable robots that target specific joints, our group has developed lightweight and soft wearable robots (“exosuits”), that assist the ankle and hip joint during (fast) loaded walking [11, 18, 21, 22], mostly for applications in first responders and soldiers for whom high speed walking is a job requirement. Exosuits use textiles to interface with the body and apply joint moments via Bowden cables pulling over the outside of the body in parallel with the muscles, using the biological skeleton to support compressive loads. Advantages of exosuits are that they have very low distal inertia and do not require precise alignment with biological joints. Recently, we achieved metabolic reductions with an autonomous version of our exosuit compared to wearing the exosuit powered-off with the equivalent mass of the actuation hardware and power source removed [11]. This version of our exosuit provided a combination of mostly positive work assistance and low negative work assistance. In contrast to earlier mentioned single-joint devices this exosuit assisted plantarflexion, hip extension and hip flexion.

Studies about devices that only assist the positive work phase of the ankle joint often motivate this actuation design choice based on the knowledge that concentric muscle work is metabolically more expensive [23], and that half of the sagittal plane lower limb positive mechanical joint work is required at the ankle [24]. In the study with the negative work phase assistance, only very low positive work assistance was provided [8]. The authors hypothesized that the reduction in metabolic rate was not due to a reduction in joint or muscle work, but rather to a reduction in isometric muscle force, while the Achilles tendon is being elongated. This claim is based on a simulation model from Umberger [25] that predicts that most of the metabolic energy consumption from the plantarflexors occurs during single stance. However, there are also recent indications that providing assistance during single stance may shift the plantarflexor muscle fascicle length toward unfavorable contractile conditions, which may disrupt the push-off phase that follows thereafter [26].

Studies that promote positive joint work assistance, and others that promote negative joint work assistance at the ankle in reducing metabolic cost are not mutually exclusive. One difficulty in reconciling these two conclusions comes from the fact that no study to date has attempted to vary the contribution of the negative work phase of the ankle joint. Therefore, the aim of the present study was to investigate the effect of negative work assistance at the ankle by varying the rate of negative work assistance de-coupled from the rate of positive work assistance. Going forward with our design approach, we chose to deliver the assistance with a version of our soft exosuit that assisted mostly the ankle. We also chose to test the effects during fast loaded walking in order to provide representative data on how to assist the ankle in first-responders or soldiers. The selection of loaded walking could lead to additional benefits for assisting negative work since biological negative joint work at the ankle is increased during loaded walking [27].

While no other study has varied negative joint work at the ankle over multiple conditions, one study from Jackson and Collins [15] contained one condition with only negative work assistance at the ankle, and compared this to a powered-off condition. Based on their dataset, we can hypothesize that varying negative work assistance will lead to an increase in metabolic cost by 8.2 J per J negative work assistance.^1^ Alternatively, based on the energy cost of eccentric muscle work reported by Margaria [23] we can hypothesize that assisting negative work at the ankle can potentially reduce metabolic cost by 1.2 J per J negative work assistance if negative work assistance would lead to an equal amount of reduction in biological work and if the efficiency of this saved biological ankle work were the same as the whole-body efficiency of downhill walking that was found by Margaria. Therefore we hypothesize that varying negative work assistance at the ankle will lead to reductions in metabolic rate between a 1.2 and 8.2 J per J negative work assistance.

## Methods

### Soft exosuit

**Fig. 1:**
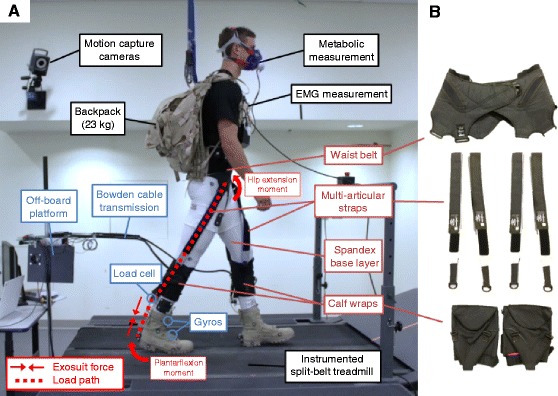
Soft exosuit, off-board actuation platform and experimental setup. **a** An off-board actuation platform generated forces based on real-time data from load cells and gyroscopes. Bowden cables transmitted the forces to the exosuit. Ground reaction force, metabolic cost and kinematics were measured while participants walked on a treadmill with a 23-kg backpack wearing the exosuit. **b** The soft exosuit consisted of a spandex base layer, a waist belt, multi-articular straps and calf wraps, creating load paths that apply plantarflexion and hip flexion assistance



The soft exosuit consisted of a spandex base layer, a waist belt, a calf wrap on each leg, and two multi-articular straps per leg connecting the calf wrap and waist belt (Fig. 1). The waist belt was similar to the one previously described in [28, 29]. We tensioned the calf wrap tightly around the wearer’s lower leg by means of Velcro straps. The multi-articular straps connected the anterior side of the waist belt with the posterior side of the calf wrap. This textile architecture creates load paths that assist the plantarflexors (e.g. the m. soleus and gastrocnemius) and the hip flexors (e.g. the m. psoas and rectus femoris) [22, 29]. We estimated the ratio between the forces in the multi-articular straps and the forces at the calf wrap from supplementary tests with load cells in the multi-articular straps in three participants (Additional files 1 and 2).

**Additional file 1:** Movie Participant walking with the exosuit in the *Powered-Off*, *Minimal* and *High* negative work rate condition. (M4 V 42521 kb)


### Off-board actuation platform

Because the goal of this study was to examine the relative effect of varying negative work assistance at the ankle joint while maintaining a fixed level of positive work assistance with a multi-articular soft exosuit, we used an off-board actuation platform similar to the one described in [20, 29–31], but with an alternative electromechanical design (Fig. 1). It consisted of two Maxon® EC motors (EC30, Maxon Motor AG, Sachseln, Switzerland; 200 W), and each motor drove a 3-cm radius pulley through a 55:1 planetary gearbox. These actuators were similar to the ones used in a fully autonomous exosuit [11] so the actuation profiles that we tested were within a range of profiles that are realistically achievable with an autonomous exosuit. We used Bowden cables to transmit the forces from the actuator to the exosuit. The end of the Bowden cable sheaths attached to the back of the calf wrap, while the inner cable extends further to the back of the heels. When the motor rotates inward, this generates forces between these two cable attachment points and also along the entire length of the load path of the multi-articular straps. The control board was a modified version of Arduino Due; it was composed of two servomotor drivers (Gold Twitter, Elmo Motion Control Ltd., Nashua, NH, USA) and two ARM-based processors (Cortex-M3, Atmel Corporation, San Jose, CA, USA), which controlled the two actuators respectively, together with CAN communication among all subsystems.

We measured real-time data from the wearer and the exosuit by means of a load cell (LTH300, Futek Advanced Sensor Technology Inc., Irvine, CA, USA) and two single-axis gyroscopes (LY3100ALH, STMicroelectronics N.V., Geneva, Switzerland) per leg. We attached two gyroscopes on the boot: one at the dorsal side of the midfoot and the other at the anterior side of the shank. We aligned the axes perpendicular to the flexion-extension axis and calculated ankle angular velocity by subtracting the angular velocities measured by both sensors.

### Control

We used a control algorithm based on an iterative force-based position controller that we described in more detail in [29]. The controller detected the beginning of the stance phase based on initial plantarflexion from the foot gyroscope. To provide negative work assistance the motor held the cable at a fixed position and passively generated force due to the effective human-suit stiffness [32, 33] as the wearer dorsiflexed. Since in normal walking the biological ankle moment is in the plantarflexion direction during the entire part of the stance phase after initial forefoot contact we know that at the ankle angular velocity zero-crossing point, the biological ankle power becomes positive. Thus, detection of ankle angular velocity zero-crossing allowed us to determine the timing for pulling on the cable to assist the positive work phase. About halfway through the push-off phase, the cable force automatically drops because the ankle joint further plantarflexes. When the controller detected this, the motor spooled the cable to prevent resistance during the swing phase.

The controller calculated exosuit power for each ankle in real time based on the product of ankle angular velocity and exosuit ankle moment (based on the load cell force and moment arm). Next, the controller calculated positive and negative exosuit work rates for each ankle respectively by integrating the positive and negative power portions and dividing them by the stride time. The controller compared measured negative and positive work rates to desired values and iteratively adjusted the pretension and active force for each ankle on a stride-by-stride basis similar to [29, 34, 35]. At the beginning of each walking condition the controller required about one minute to reach the desired rate of negative work assistance.

It should be noted that due to the multi-articular strap the exosuit also passively applied positive and negative work at the hip joint. The amount of work at the hip resulted passively from the forces applied at the ankle and the amount by which the straps were tightened. It was not directly controlled since we did not have separate actuation at the hip.

### Participants

Eight healthy male participants (26.3 ± 4.7 y, 79.9 ± 9.5 kg, 1.78 ± 0.06 m) who were experienced load carriers or endurance athletes participated in this study. The Harvard Medical School Committee on Human Studies approved the study and the participants provided written informed consent. The participants whose images appear in the manuscript have provided written consent for the publication of their images according to the policies of the Journal of NeuroEngineering and Rehabilitation.

### Protocol

Participants attended two experimental sessions, a training and a testing session that were 2 to 7 days apart. Appropriate sizes of the exosuit components were fitted during training. After donning the system, participants walked on a treadmill at 1.5 m s^−1^ while carrying a load of 23-kg and experienced all of the different actuation conditions for 8 min each, for a total of 32 min of training [36]. During the testing session participants completed the same protocol as in the training plus an additional 8-min baseline condition. In this condition, called *Powered-Off*, the participants wore the exosuit and the backpack but the cables were slack such that they had close to zero tension so that no moments were applied to the joints. We randomized the order of the conditions.

### Experimental conditions

**Fig. 2:**
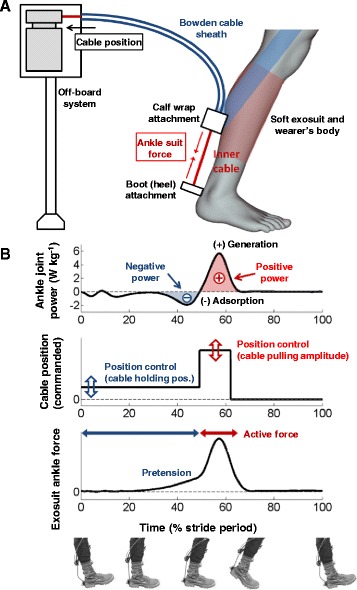
Controller. **a** A schematic diagram of the actuation hardware and control of the soft exosuit. **b** The methodology for controlling the pretension and the active force. The plots illustrate the force, the commanded cable position and the ankle plantarflexion angle within a gait cycle, respectively

During the training session, we determined the highest positive work rate that we could consistently deliver to both legs in the four active conditions (The positive work rate is shown as the positive part of the power curve in Fig. 2b). In the *Minimal* negative work rate condition, the control provided the minimal amount of pretension (Fig. 2c, d) that was required to make sure that the cable between the boot attachment and calf wrap attachment (Fig. 2a) was taut at the beginning of the positive work assistance phase such that we could maintain the rate of positive work assistance. This minimal cable force was required for stable behavior of the controller. In *Low*, *Medium* and *High* we set the desired negative work rates (i.e. part of the power curve that is marked in Fig. 2b) for each ankle respectively at 10, 20 and 30% of the desired positive work rate per leg.

### Measurements

During the testing session we measured respiratory gases by means of a computerized O_2_-CO_2_ analyzer and flow meter (K4b^2^, COSMED, Rome, Italy) during a 4-min standing trial and during the walking conditions. We recorded 3D kinematics using motion capture (Vicon, Oxford Metrics, Oxford, UK). We attached reflective markers to the boots, shank, thigh, pelvis, trunk and Bowden cables attachments. We acquired ground reaction force data from split-belt instrumented treadmill (Bertec Corporation, Columbus, OH, USA).

### Data processing

We calculated metabolic rate by means of the Brockway equation [37] and averaged the last 2 min of each trial. We averaged only the last two minutes in order to allow sufficient time for the exosuit actuation to stabilize and for metabolic rate to reach steady state. We filtered raw kinematic and ground reaction force data using a zero-lag, low-pass, fourth-order Butterworth filter with a cut-off frequency between 8 Hz and 15 Hz, as determined by preliminary residual analysis. We calculated sagittal plane joint kinetics and kinematics using Visual3D (C-motion Inc., Rockville, MD, USA). We calculated the exosuit moments based on a moment arm determined from markers on the exosuit load path and joint axes. We calculated biological joint kinetics by subtracting the exosuit joint kinetics from the total external joint kinetics [7, 8, 11, 15, 16, 19, 20]. We calculated center-of-mass displacement by dividing total ground reaction force by body mass and integrating over time [38]. We calculated center-of-mass power for each leg by taking the dot product of center-of-mass velocity and the individual leg ground reaction force [39]. We determined heel contact and toe off times based on ground reaction forces and kinematics using the automatic gait detection algorithm from Visual3D [40]. We averaged kinematics and kinetics of 10 strides of the last minute of each walking condition and plotted the results versus stride percentage. After each condition we gave the participants a questionnaire in which we asked them to score the perceived assistance and perceived comfort on a VAS scale similar to [34]. We excluded data from one participant because we noticed that the waist belt slipped during testing, which resulted in the system malfunctioning and large standard deviations in the physiological data. In addition, for two participants we were unable to provide the desired negative work rate in *High*, due to the participants’ specific gait kinematics and the performance limitations of the off-board actuation platform. For these two participants we only collected and analyzed data from three active conditions.

### Statistics

We used mixed-model, two-factor ANOVA with participants as random-effect to test the effect of of negative exosuit ankle work rate. To evaluate differences between the conditions we performed repeated-measures ANOVA. If the *p*-value was equal to or lower than 0.05, we conducted pairwise comparisons between *Powered-Off* and the active conditions with paired t-tests with Šídák-Holm correction for multiple testing. In order to evaluate where significant effects were situated in time with respect to the actuation period, we performed statistics for all frames of the stride cycle and visually represented results in colorbars above time series charts similar to [41, 42]. We also conducted the same statistical tests on relevant single-value metrics (e.g. metabolic rate). We reported all inter-subject variabilities as standard errors of the means.

## Results

### Exosuit mechanics

**Fig. 3:**
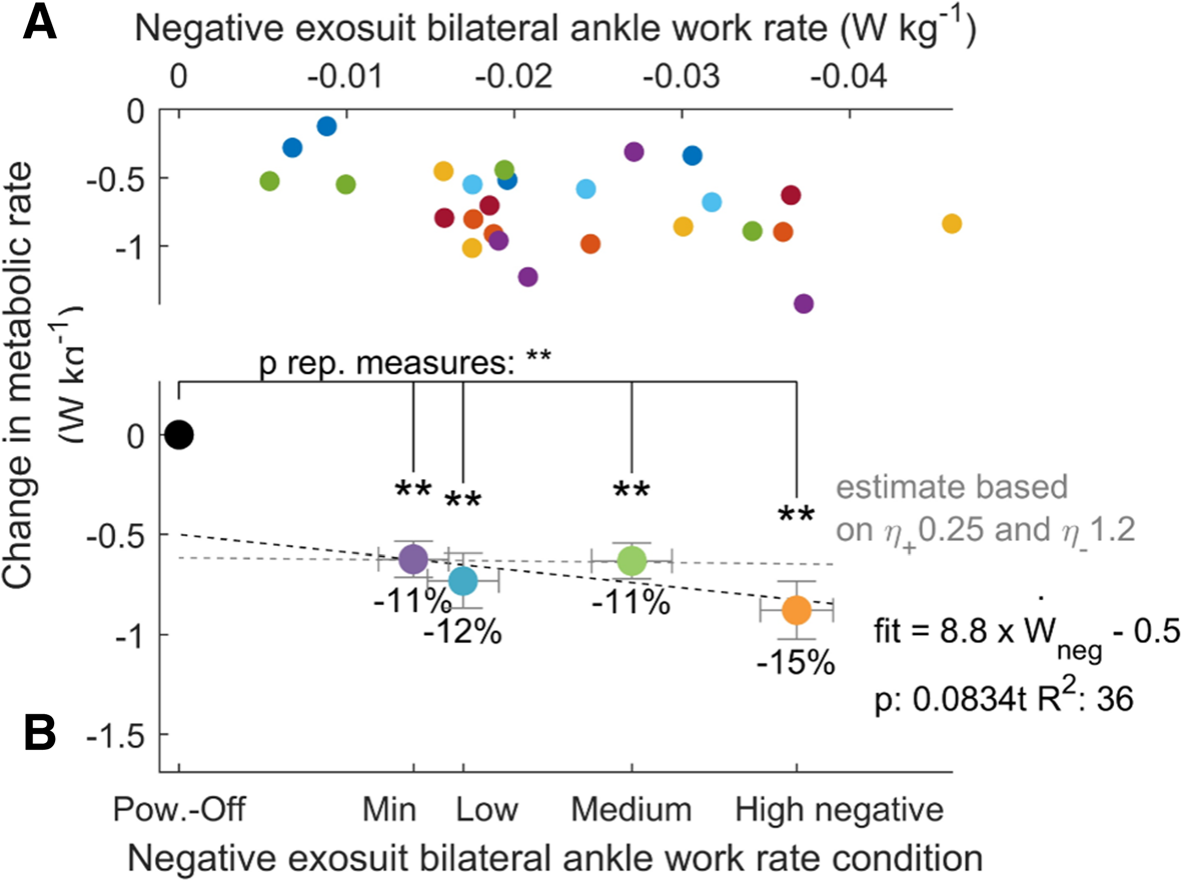
Change in metabolic rate versus negative exosuit bilateral ankle work rate. **a** Individual data. Colors are different participants. **b** Average for each condition. Error bars are s.e.m. Percentages are percent metabolic reduction. Dashed black line indicates linear fit from mixed-model ANOVA. *Dashed grey line* indicates expected trend assuming exosuit positive and negative work efficiencies corresponding to the reported efficiency of biological muscles [23]. *Brackets* indicate pairwise differences versus *Powered-Off*. Black dot represents *Powered-Off*. ** is *p* ≤ 0.01, * is *p* ≤ 0.05

**Fig. 4:**
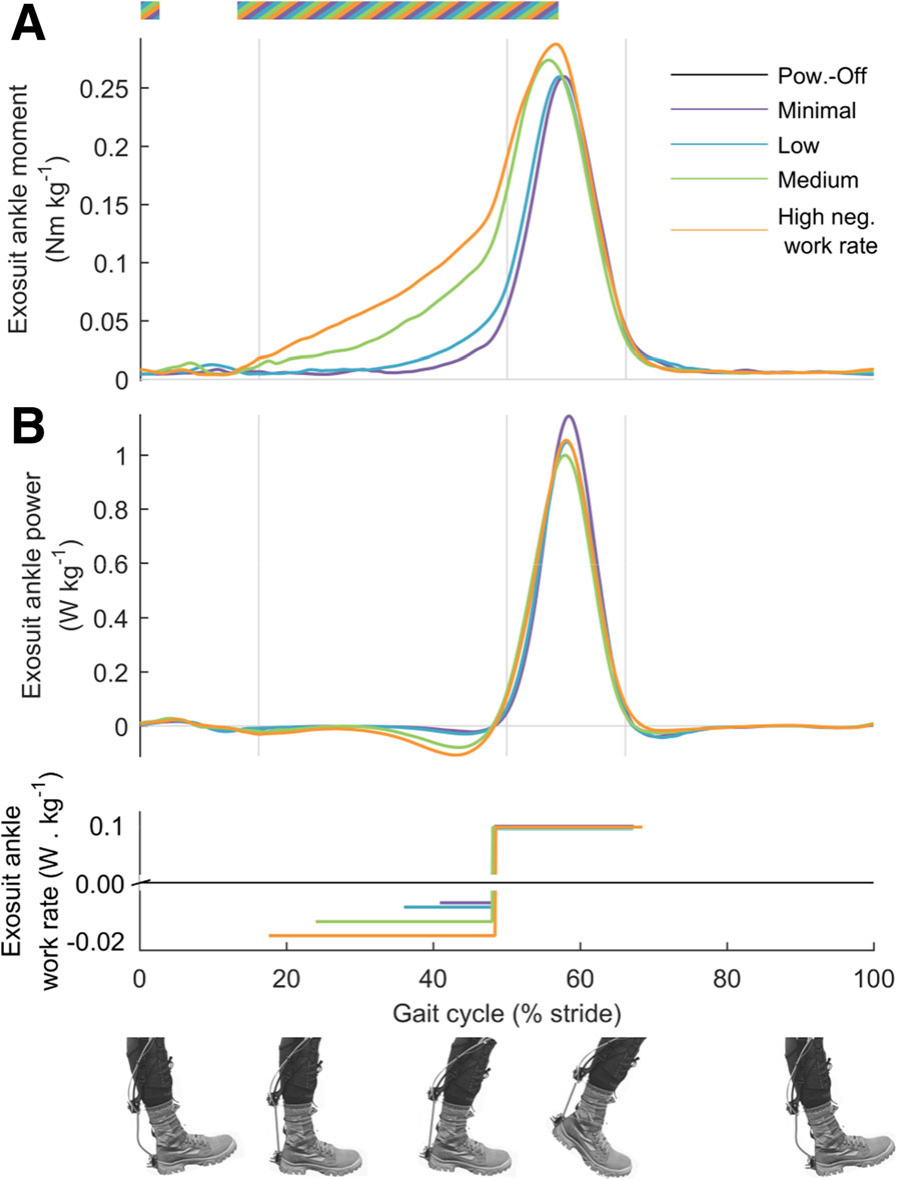
Ankle actuation. **a** Exosuit ankle moment. **b** Exosuit ankle power. *Colored lines* represent average time series in the four active conditions. *Lower panel* shows duration and rate of negative and positive work assistance per leg in conditions with corresponding colors. Grey vertical lines delimit double and single stance phases. Multi-colored bars indicate significant result of mixed-model ANOVA with the rate of negative work assistance as fixed-effect (*p* ≤ 0.05). Related metric plots can be found in Additional file 6

Average negative work rates of the left plus right exosuit ankle in *Minimal*, *Low*, *Medium* and *High* respectively were - 0.015 ± 0.005, − 0.016 ± 0.007, − 0.027 ± 0.006 and - 0.037 ± 0.006 W kg^−1^ (Fig. 3). To achieve this range of negative work rates exosuit ankle pretension moments increased from 0.04 ± 0.01 Nm kg^−1^ in *Minimal* up to 0.16 ± 0.01 Nm kg^−1^ in *High* (*p* = 6 · 10^−8^, mixed-model ANOVA with negative exosuit work as fixed factor showing significant relationship between exosuit ankle pretension moment and negative exosuit ankle work rate, Fig. 4a). As a result of the changes in pretension force and ankle angle, the effective angular stiffness of the exosuit at the ankle during the dorsiflexion phase increased from 0.007 to 0.019 Nm (kg °)^−1^ (*p* = 0.003) from *Minimal* to *High* (Additional file 3). The positive exosuit bilateral ankle work rate of the active conditions was 0.19 ± 0.01 W kg^−1^ and stayed constant independent from exosuit ankle negative work rate (*p* = 0.22, Fig. 4b). However, higher negative work rate conditions required higher peak exosuit moment at the ankle to achieve the same positive work rate (*p* = 8 · 10^−7^, Fig. 4a). Peak moment increased from 0.30 ± 0.10 Nm kg^−1^ in *Minimal* to 0.35 ± 0.20 Nm kg^−1^ in *High*.

**Fig. 5:**
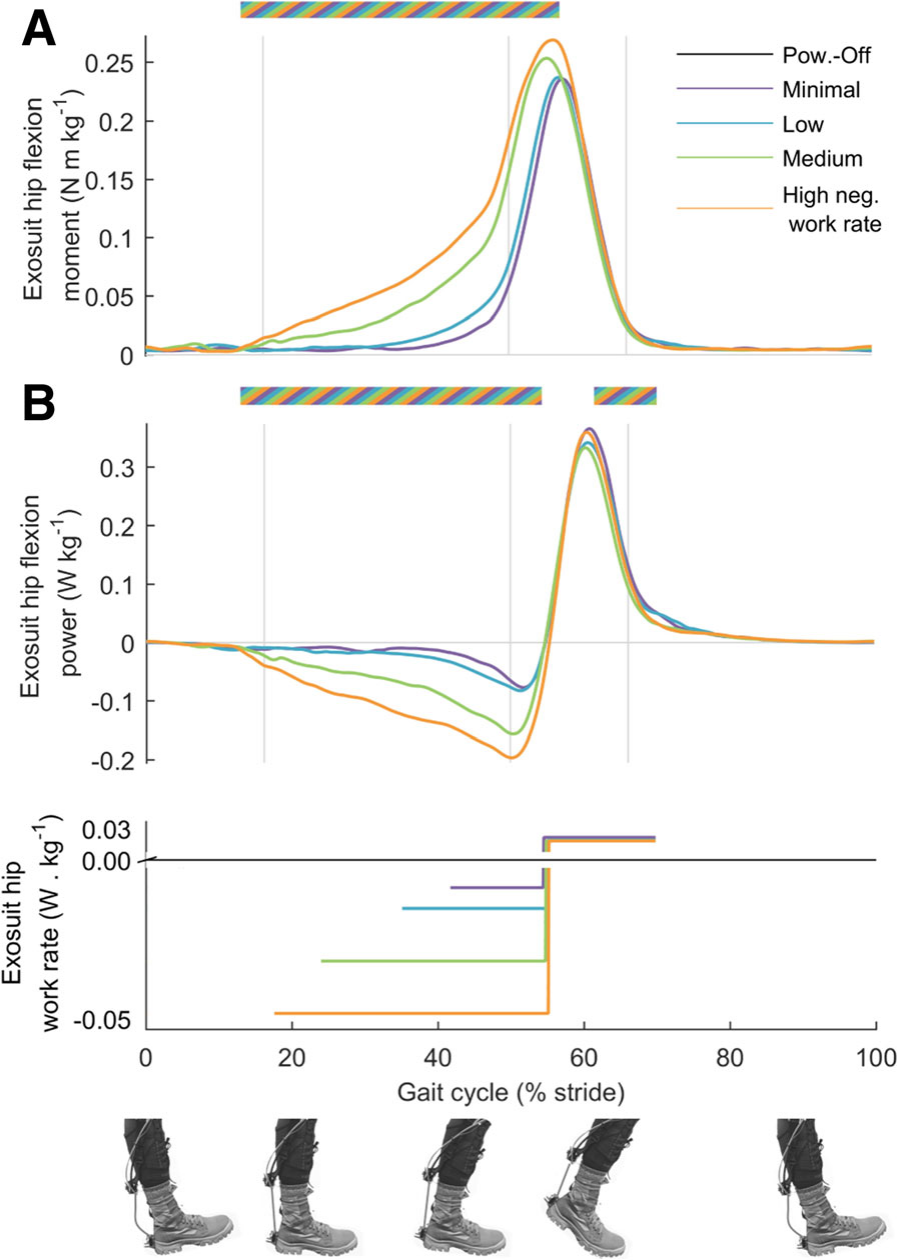
Hip actuation. **a** Estimated exosuit hip moment. **b** Estimated exosuit hip power. *Colored lines* represent average time series from *left* and *right* leg in conditions with different rates of negative work assistance. *Lower panel* shows duration and rate of negative and positive work assistance per leg in conditions with corresponding colors. *Grey vertical lines* delimit double and single stance phases. *Multi-colored bars* indicate significant result of mixed-model ANOVA with the rate of negative work assistance as fixed-effect (*p* ≤ 0.05). Related metric plots can be found in Additional file 7

In supplementary tests we used an additional load cell on the proximal attachment of the multi-articular strap and calculated the ratio of the strap forces at the hip versus strap forces at the ankle. On average, this ratio was 0.65 (Additional file 1). Based on the estimated ratio of load distribution between the calf wrap and waist belt we estimated that the negative exosuit hip work rate changed from - 0.020 ± 0.002 to - 0.097 ± 0.016 W kg^−1^ with increasing negative exosuit ankle work rate (*p* = 2 · 10^−12^, Fig. 5). Estimated positive exosuit hip work rate did not remain constant but decreased slightly from 0.068 ± 0.013 to 0.064 ± 0.014 W kg^−1^ with increasing negative exosuit ankle work rate (*p* = 0.046).

### Metabolic rate

Metabolic rates of walking minus resting in *Minimal*, *Low*, *Medium* and *High* respectively were 5.21 ± 0.20, 5.10 ± 0.16, 5.20 ± 0.21 and 5.00 ± 0.24 W kg^−1^. Metabolic rate of walking minus resting in *Powered-Off* was 5.84 ± 0.20 W kg^−1^. In all the active conditions metabolic rate was significantly lower than in *Powered-Off* (*p* ≤ 5 · 10^−4^, paired t-tests). Reductions in metabolic rate compared to *Powered-Off* in *Minimal*, *Low*, *Medium* and *High* respectively were 0.62 ± 0.09, 0.73 ± 0.14, 0.63 ± 0.09, 0.88 ± 0.17 W kg^−1^. Percent reductions Compared to *Powered-off* were 11 ± 1, 12 ± 2, 11 ± 2 and 15 ± 3% (Fig. 3b).

There was no significant effect of negative exosuit ankle work rate on reduction in metabolic rate versus *Powered-Off* but there was a trend toward significance (*p* = 0.083). The average efficiency of negative exosuit ankle work assistance is visualized by the slope of this trend in Fig. 3b which amounts to 8.8 W reduction in metabolic rate per W negative work rate from both exosuit ankles. The average efficiency of positive exosuit ankle work assistance can be derived from the intercept value of this trend with a vertical line through zero negative work rate on the horizontal axis, divided by the positive exosuit ankle work rate. This efficiency of positive work assistance was 2.7 W reduction in metabolic rate per W total positive exosuit ankle work rate.

### Kinematics

**Fig. 6:**
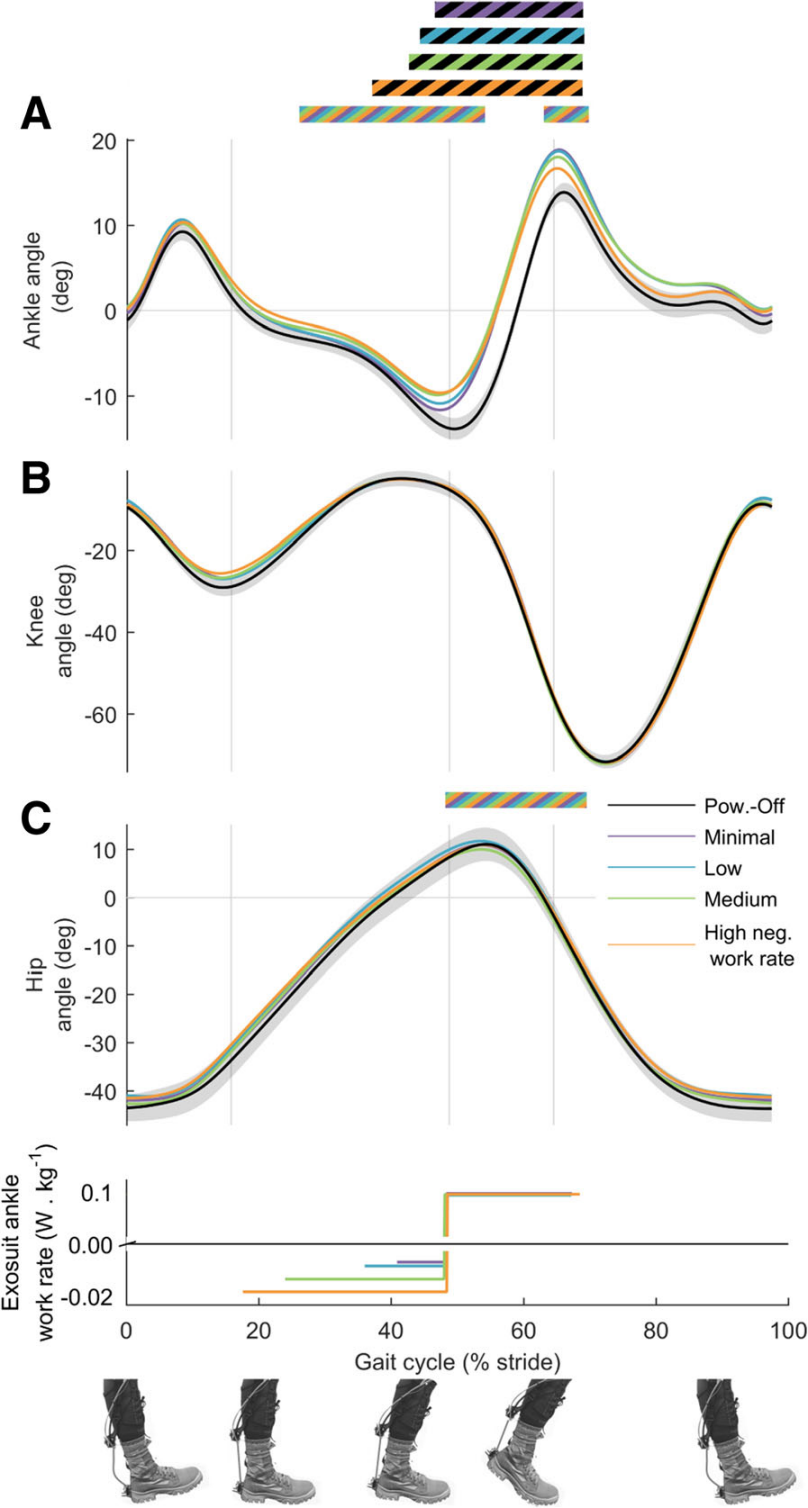
Joint kinematics. **a** Ankle angle. **b** Knee angle. **c** Hip Angle. *Colored lines* represent average time series from *left* and *right* leg in conditions with different rates of negative work assistance. *Lower panel* shows duration and rate of negative and positive work assistance per leg in conditions with corresponding colors. *Black line* is *Powered-Off* and shaded areas represent standard error. *Grey vertical lines* delimit double and single stance phases. Bi-colored bars (black and other color) above time series, indicate periods with significant pairwise difference versus *Powered-Off* of the condition with the corresponding color (*p* ≤ 0.05). *Multi-colored bars* indicate significant result of mixed-model ANOVA with the rate of negative work assistance as fixed-effect (*p* ≤ 0.05). Related metric plots can be found in Additional file 8

Increasing negative exosuit ankle work rate led to reduced dorsiflexion angle around mid-stance (*p* ≤ 0.05 from 29 to 53% of the stride cycle, Fig. 6a) and also led to reduced peak plantarflexion angle at the end of push-off (*p* = 0.008, ANOVA). In all of the active conditions plantarflexion started earlier than in *Powered-Off* (*p* ≤ 0.05 from 50 to 63% of the stride cycle).

In *Minimal* there was less maximal knee flexion during early stance than in the *Powered-Off* (*p* = 0.030, Fig. 6b). Increasing negative exosuit ankle work rate led to reduced maximum hip extension (*p* = 0.015, Fig. 6c).

### Biological joint kinetics

**Fig. 7:**
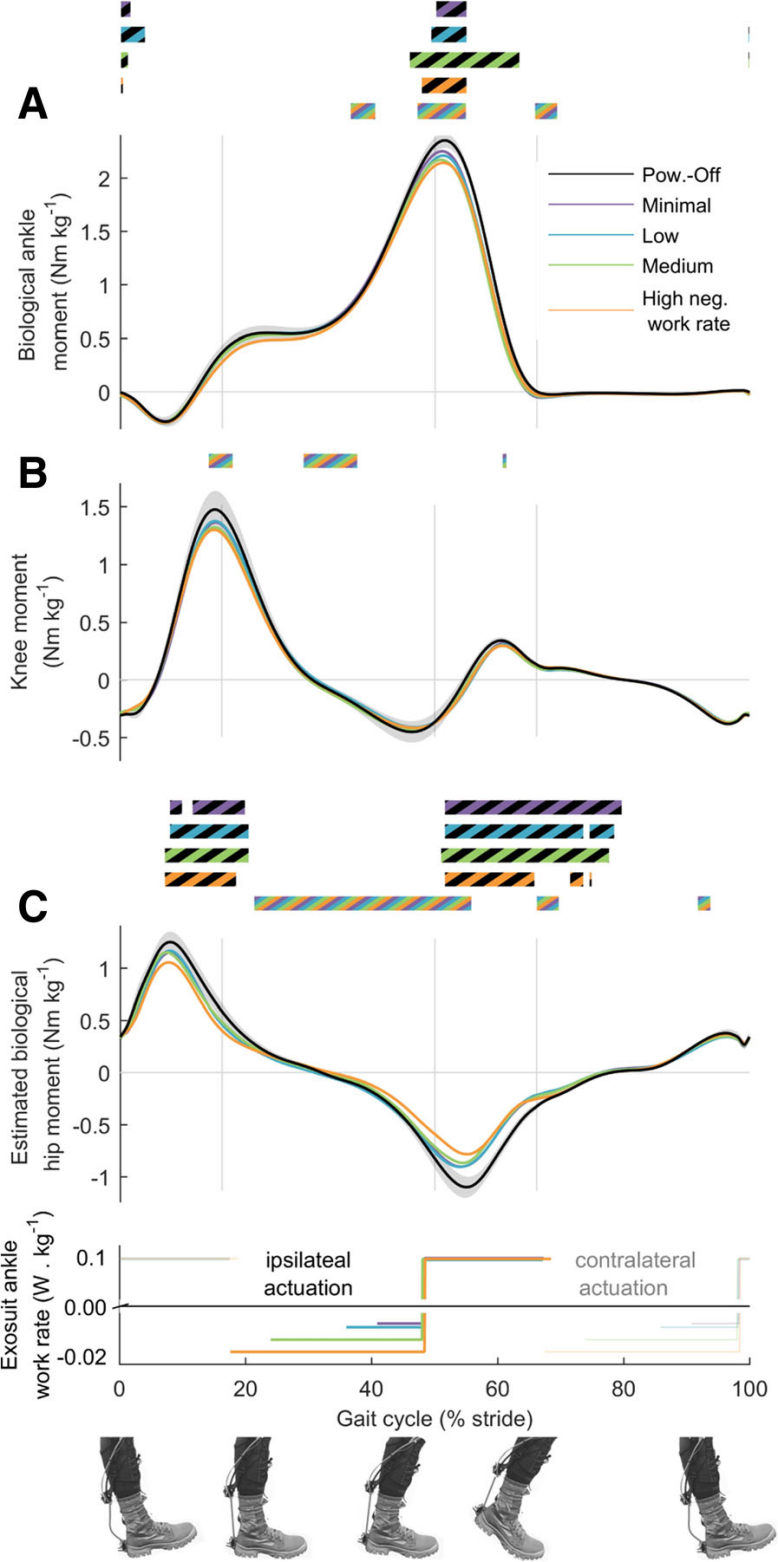
Biological joint moments. **a** Biological ankle moment. **b** Knee moment. **c** Estimated biological hip moment. *Colored lines* represent average time in conditions with different rates of negative work assistance. *Lower panel* shows duration and rate of negative and positive work assistance per leg in conditions with corresponding colors. Transparent lines indicate opposite leg actuation period. *Black line* is *Powered-Off* and shaded areas represent standard error. *Grey vertical lines* delimit double and single stance phases. Bi-colored bars (black and other color) above time series, indicate periods with significant pairwise difference versus *Powered-Off* from the condition with the corresponding color (*p* ≤ 0.05). Multi-colored bars indicate significant result of mixed-model ANOVA with the rate of negative work assistance as fixed-effect (*p* ≤ 0.05). Related metric plots can be found in Additional file 9

Peak biological ankle moment decreased with increasing negative exosuit ankle work rate (*p* = 4 · 10^−4^, Fig. 7a). In all active conditions peak biological ankle moment was reduced compared to *Powered-Off* (*p* ≤ 0.019).

Estimated biological hip extension and flexion moment decreased with increasing negative exosuit ankle work rate during early stance and the beginning of push-off (*p* ≤ 0.05 from 24 to 54% of the stride cycle, Fig. 7c). In all active conditions, estimated biological hip flexion moment was reduced compared to *Powered-Off* during the push-off phase of the ipsilateral leg (*p* ≤ 0.05 from 13 to 18% of the stride period), as well as during the push-off phase of the contralateral leg (*p* ≤ 0.05 from 54 to 64% of the stride period).

**Fig. 8:**
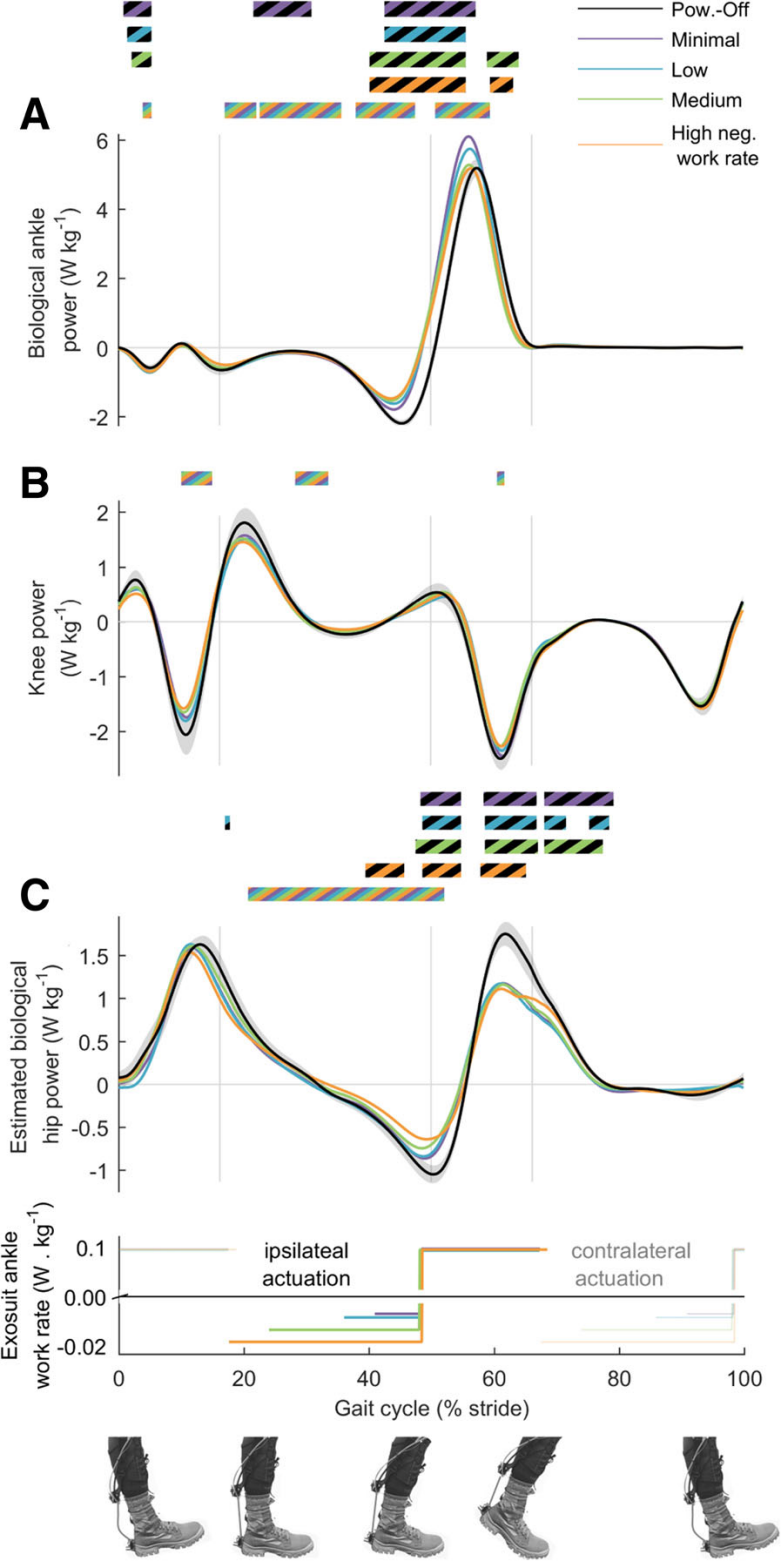
Biological joint powers. **a** Biological ankle power. **b** Knee power. **c** Estimated biological hip power. *Colored lines* represent average time series from *left* and *right* leg in conditions with different rates of negative work assistance. *Lower panel* shows duration and rate of negative and positive work assistance per leg in conditions with corresponding colors. Transparent lines indicate opposite leg actuation period. *Black line* is *Powered-Off* and shaded areas represent standard error. *Grey vertical lines* delimit double and single stance phases. Bi-colored bars (black and other color) above time series, indicate periods with significant pairwise difference versus *Powered-Off* from the condition with the corresponding color (*p* ≤ 0.05). *Multi-colored bars* indicate significant result of mixed-model ANOVA with the rate of negative work assistance as fixed-effect (*p* ≤ 0.05). Related metric plots can be found in Additional file 10

Negative biological ankle power decreased with increasing negative exosuit ankle work rate during early stance (*p* = 7 · 10^−6^, Fig. 8a), and positive biological ankle power during the push-off phase (*p* = 9 · 10^−6^) decreased with increasing negative exosuit ankle work rate. In all the active conditions the transition from negative to positive biological ankle power occurred earlier than in *Powered-Off* due to earlier onset of plantarflexion (*p* ≤ 0.05 from 43 to 53% of the stride cycle).

The estimated biological hip power decreased with increasing negative exosuit ankle work rate, from 23 to 50% of the stride cycle (*p* ≤ 0.05). Both negative and positive estimated biological hip power during the push-off phase showed significant reductions compared to *Powered-Off* in all the active conditions (*p* ≤ 0.05, Fig. 8c).

### Total body kinetics

**Fig. 9:**
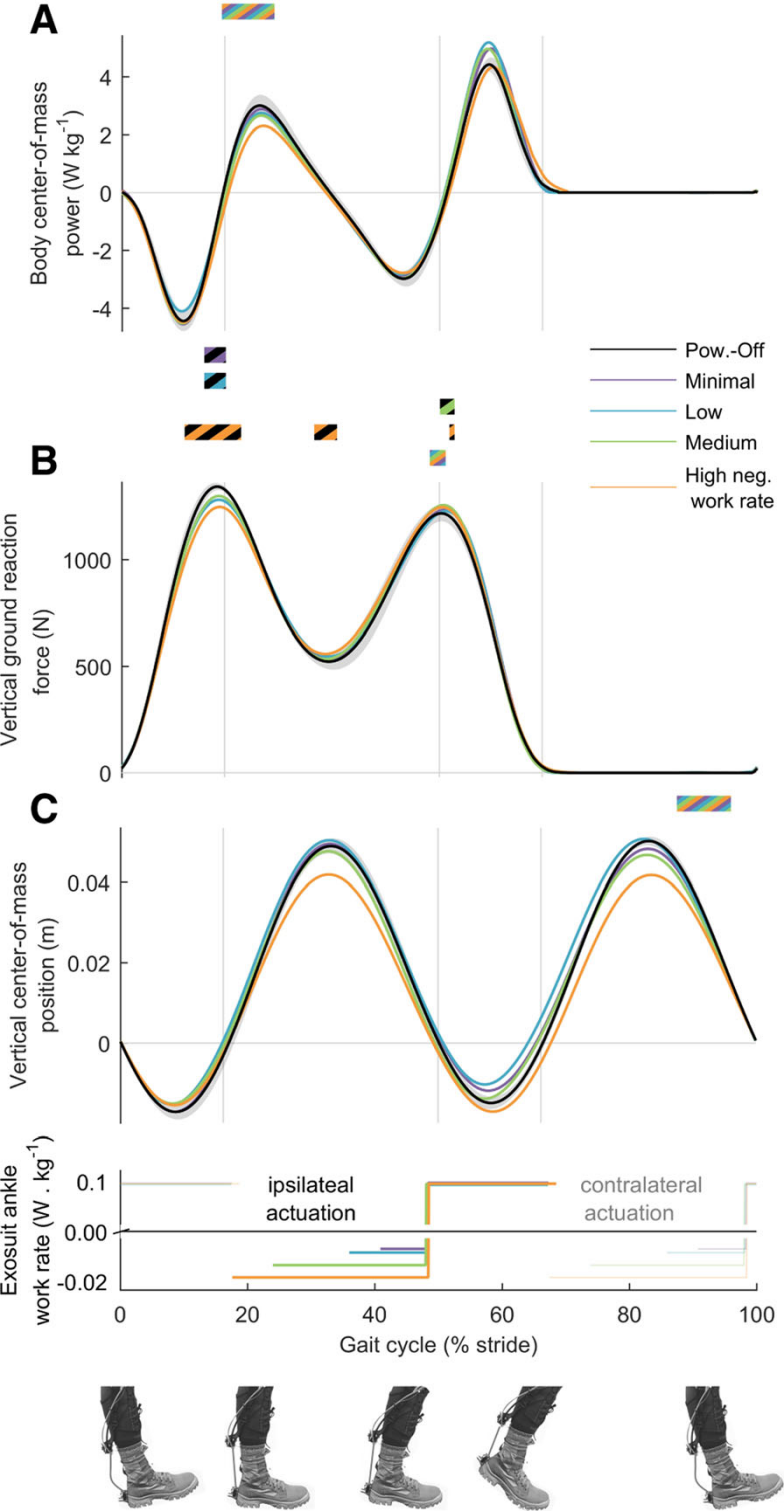
Total body kinematics and kinetics. **a** Vertical center-of-mass position (**b**) Center-of-mass power, (**c**) Vertical ground reaction force. *Colored lines* represent average time series from *left* and *right* leg in conditions with different rates of negative work assistance. *Lower panel* shows duration and rate of negative and positive work assistance per leg in conditions with corresponding colors. Transparent lines indicate opposite leg actuation period. *Black line* is *Powered-Off* and shaded areas represent standard error. *Grey vertical lines* delimit double and single stance phases. Bi-colored bars (black and other color) above time series, indicate periods with significant pairwise difference versus *Powered-Off* from the condition with the corresponding color (*p* ≤ 0.05). Multi-colored bars indicate significant result of mixed-model ANOVA with the rate of negative work assistance as fixed-effect (*p* ≤ 0.05). Related metric plots can be found in Additional file 4

Total body center-of-mass rebound work decreased with increasing negative exosuit ankle work rate (*p* = 2 · 10^−6^, Fig. 9a). The first peak of the vertical ground reaction force was reduced compared to *Powered-Off* in all of the active conditions (*p* ≤ 0.023, Additional file 4).

## Discussion

All the active conditions significantly reduced metabolic rate compared to *Powered-Off* by 11 to 15%. There was a trend showing increasing reduction in metabolic rate with increasing negative work assistance (Fig. 3b). This trend followed an average slope of 8.8 J metabolic reduction per J negative work assistance. This slope was different from the expected range based on ankle work which was between slope of 8 J metabolic increase per J negative work [15] and 1.2 J metabolic reduction per J negative work [23]. However, the slope of the trend in our study was not significant.

There are different possible reasons as to why there was no significant effect of negative work assistance on metabolic rate. The negative work assistance provided in *High* was only about half the negative work in the optimal stiffness condition in the study from Collins et al. [8]. In this study we were limited in our ability to provide higher negative work due to the fact that the controller used maintained the ankle cable at a fixed length during the pretension phase. Additionally, due to the fast walking velocity of our study (1.5 m s^−1^), the duration of the dorsiflexion phase was relatively short, which limits the percentage of the gait cycle where negative work can be applied [43, 44]. Even if the negative work assistance would have reduced metabolic rate with the same efficiency as eccentric contractions in biological muscles [23], the range of negative work assistance that we applied would only have led to a change in metabolic rate of 4%—close to the observed average difference between *Minimal* and *High*. Similar to the study from Collins et al. [8] we also found that peak dorsiflexion decreased with increasing negative exosuit ankle work rate. This could potentially put the plantar flexors in disadvantageous contractile conditions for push off by reducing Achilles tendon stretch [8, 45].

Conversely, there are different possible explanations as to why we still found a small trend toward higher reduction in metabolic rate with increasing negative work assistance. We found significant effects of negative exosuit ankle work resulting increases in estimated exosuit hip power and moment and reductions in estimated biological hip power during the negative work assistance phase. These are likely the effects of the force transfer from the multi-articular straps or indirect effects of plantarflexion assistance [17, 45] or a combination of both. We also found reductions in biological ankle peak power and moment with increasing negative work assistance probably due to the fact that the participants and the controller generated higher peak force in increasing negative work assistance conditions. Finally, we found higher reductions in the center-of-mass rebound work with increasing negative work assistance.

One limitation for interpreting the effect of negative work assistance at the ankle is that we did not keep the hip assistance constant. While we were able to rigorously control the exosuit ankle work and to maintain the positive portion constant, this also resulted in changes in the exosuit hip power, though to a lesser extent than in the ankle actuation. In this version of the exosuit, the use of the multi-articular strap that couples ankle actuation to hip actuation was a necessary compromise in order to keep the calf wrap from slipping. This is a common challenge of parameter sweeps with exoskeletons. As control schemes improve, we are now able to very rigorously manipulate certain parameters of interest and keep others constant. However, it will never be possible to keep all parameters constant, even with single-joint exoskeletons, simply because certain parameters inherently vary with the parameter of interest. Moreover, we only measured the multi-articular strap force distribution in tests that were separate from the biomechanical and physiological evaluation reported here so the reported exosuit hip moments and powers are only estimations. Further, we calculated the changes in metabolic rate compared to a *Powered-Off* condition and not compared to a no-suit condition; thus, the effect of wearing the exosuit components is evaluated in the results. This was done to simplify the protocol and reduce the time between relative metabolic comparisons such that subjects did not have to stop and doff the exosuit between conditions. It is currently unknown how such a comparison would change the results, but due to the lightweight nature of the exosuit components (about one kg total distributed on both legs, Additional file 5), we expect that the difference would be small. For this reason, however, the augmentation factor proposed by Mooney et al. [9, 10] was not calculated in this study due to the lack of a strict no-suit condition.

The derived efficiency of positive work assistance of 2.7 J metabolic reduction per J mechanical work falls within the range of ratios reported in rigid ankle exoskeletons studies (1.6 in [16], 2.7 in [15], 3.9 in [14], 4.7 in [7]). Possible explanations for the benefits of positive work assistance can be found in differences in biomechanics versus *Powered-Off* that occurred during the positive work assistance phase. For example, we found reductions in biological ankle moment during push-off with respect to *Powered-Off*. We also found reductions in biological hip flexion power and biological hip extension and flexion moment during this phase.

The observed benefits of positive work assistance together with the absence of a significant metabolic benefit of negative work assistance suggest that for autonomous applications of this exosuit, focusing on ankle positive power assistance should be considered and there may be small additional benefits, and no major biomechanical disadvantages of also providing assistance during the ankle negative power phase. Additional considerations could also be on the actuation requirements to realize the intended assistance and how this would affect system and battery mass. It is likely that these results are specific to the architecture of the exosuit used here and different results could be found when assisting different joints with other exosuits architectures or rigid exoskeletons or with different carried loads. It is also possible that a different result would be found if the negative and positive work assistance would be applied with different force profile shapes. More research is needed to provide a definitive answer on when negative work assistance is beneficial or detrimental.

## Conclusions

This study describes how to provide both negative and positive work assistance at the ankle with a soft exosuit during loaded walking by providing a low pretension force at the heel while the ankle dorsiflexes and actively pulling as soon as the ankle starts plantarflexing. All the active conditions significantly reduced metabolic rate on average by 11 to 15% versus *Powered-Off*. We found a trend toward more reduction in metabolic rate with increasing negative work assistance but this was not significant. However, we did see significant benefits of negative work assistance on biological ankle and hip joint kinetics which could explain the trend toward significant metabolic reduction. Positive work assistance led to high reductions in metabolic rate relative to the provided positive work. The non-significant trend of increasing negative work assistance with increasing reductions in metabolic rate motivates the value in further studies on the relative effects of negative and positive work assistance. In particular, there may be benefit in investigating varying negative work over a greater range or in varying negative work assistance without providing positive work assistance.

## Additional files

Additional file 2: Multi-articular strap hip versus ankle force ratio. Average force ratio from separate tests in three participants with an additional load cell on the proximal attachment of the multi-articular straps. Error bars are s.e.m. (PDF 36 kb)

Additional file 3: Joint moments versus joint angle relationships. **(A)** Ankle joint moment versus ankle angle. **(B)** Knee moment versus knee angle. **(C)** Hip moment versus hip angle. Colored lines represent average time in conditions with different rates of negative work assistance. Black line is Powered-Off and shaded areas represent standard error. (PDF 2 kb)

Additional file 4: Total body kinematics and kinetics timeseries. **(A)** Center-of-mass rebound work rate. **(B)** Vertical ground reaction force first peak. Dots are condition averages. Error bars are s.e.m. Dashed black line indicates linear fit from mixed-model ANOVA. Brackets indicate pairwise differences versus *Powered-Off*. Black dot represents *Powered-Off* reference condition. ** is *p* ≤ 0.01, * is *p* ≤ 0.05. (PDF 570 kb)

Additional file 5: Exosuit weight split out by components. (PDF 13 kb)

Additional file 6: Ankle actuation metrics. **(A)** Exosuit ankle pretension force. Exosuit ankle pretension force is defined as the peak force at the end of the negative work phase. **(B)** Peak exosuit ankle force. **(C)** Positive exosuit ankle work rate. Dots are condition averages. Error bars are s.e.m. Dashed black line indicates linear fit from mixed-model ANOVA. Brackets indicate pairwise differences versus *Powered-Off*. Black dot represents *Powered-Off* reference condition. ** is *p* ≤ 0.01, * is *p* ≤ 0.05. (PDF 870 kb)

Additional file 7: Hip actuation metrics. **(A)** Exosuit hip pretension force. Exosuit hip pretension force is defined as the peak force at the end of the negative work phase. **(B)** Peak exosuit hip force. **(C)** Negative exosuit hip work rate. **(D)** Positive exosuit hip work rate. Dots are condition averages. Error bars are s.e.m. Dashed black line indicates linear fit from mixed-model ANOVA. Brackets indicate pairwise differences versus *Powered-Off*. Black dot represents *Powered-Off* reference condition. ** is *p* ≤ 0.01, * is *p* ≤ 0.05. (PDF 1 mb)

Additional file 8: Joint kinematics metrics. **(A)** Peak dorsiflexion. **(B)** Peak plantarflexion. **(C)** Maximum knee flexion during load acceptance. **(D)** Peak knee extension. **(E)** Peak hip extension. **(F)** Peak hip flexion. Dots are condition averages. Error bars are s.e.m. Dashed black line indicates linear fit from mixed-model ANOVA. Brackets indicate pairwise differences versus *Powered-Off*. Black dot represents *Powered-Off* reference condition. ** is *p* ≤ 0.01, * is *p* ≤ 0.05. (PDF 1 mb)

Additional file 9: Biological joint moment metrics. **(A)** Biological ankle peak plantarflexion moment. **(B)** Estimated biological hip extension impulse. **(C)** Estimated biological hip flexion impulse. Dots are condition averages. Error bars are s.e.m. Dashed black line indicates linear fit from mixed-model ANOVA. Brackets indicate pairwise differences versus *Powered-Off*. Black dot represents *Powered-Off* reference condition. ** is *p* ≤ 0.01, * is *p* ≤ 0.05. (PDF 891 kb)

Additional file 10: Biological joint work metrics. **(A)** Biological ankle A1 work rate. **(B)** Biological ankle A2 work rate. **(C)** Estimated biological hip H1 work rate. **(D)** Estimated biological hip H2 work rate. **(E)** Estimated biological hip H3 work rate. Dots are condition averages. Error bars are s.e.m. Dashed black line indicates linear fit from mixed-model ANOVA. Brackets indicate pairwise differences versus *Powered-Off*. Black dot represents *Powered-Off* reference condition. ** is *p* ≤ 0.01, * is *p* ≤ 0.05. (PDF 1 mb)

## Footnotes

1 We calculated the ratio of change in metabolic rate per unit negative work rate assistance based on the dataset from Jackson and Collins [15] that was made available for download in [46]. From their dataset, we calculated the increase in metabolic cost in the condition where only negative work was provided by subtracting the metabolic rate in the powered-off condition from the condition in which only negative work was provided. Then, we calculated the rate of negative work that was provided in that condition by integrating the exoskeleton ankle joint power over time and dividing by stride time. Finally, we divided the increase in metabolic rate by the negative exoskeleton ankle work rate to obtain an individual ratio for every participant. The mean of this ratio is 8.2 J increase in metabolic rate per J negative work.

## References

[1] Caron RR, Lewis CL, Saltzman E, Wagenaar RC, Holt KG (2015). Musculoskeletal stiffness changes linearly in response to increasing load during walking gait. J Biomech.

[2] Bastien GJ, Willems PA, Schepens B, Heglund NC (2005). Effect of load and speed on the energetic cost of human walking. Eur J Appl Physiol.

[3] Knapik J, Reynolds K, Harman E (2004). Soldier load carriage: historical, physiological, biomechanical, and medical aspects. Mil Med.

[4] Walsh CJ, Paluska D, Pasch K, Grand W, Valiente A, Herr H. Development of a lightweight, underactuated exoskeleton for load-carrying augmentation. Orlando: Int. Conf. Robot. Autom.; 2006. p. 3485–3491. http://ieeexplore.ieee.org/abstract/document/1642234/.

[5] Gregorczyk KN, Hasselquist L, Schiffman JM, Bensel CK, Obusek JP, Gutekunst DJ (2006). Effects of a lower-body exoskeleton device on metabolic cost and gait biomechanics during load carriage. Ergonomics.

[6] James Walsh C, Endo K, Herr H (2007). A quasi-passive leg exoskeleton for load carrying augmentation. Int J Humanoid Robot.

[7] Malcolm P, Derave W, Galle S, De Clercq D (2013). A simple exoskeleton that assists plantarflexion can reduce the metabolic cost of human walking. PLoS One.

[8] Collins SH, Wiggin MB, Sawicki GS (2015). Reducing the energy cost of human walking using an unpowered exoskeleton. Nature.

[9] Mooney LM, Rouse EJ, Herr HM (2014). Autonomous exoskeleton reduces metabolic cost of human walking. J. Neuroeng. Rehabil..

[10] Mooney LM, Rouse EJ, Herr HM (2014). Autonomous exoskeleton reduces metabolic cost of human walking during load carriage. J. Neuroeng. Rehabil..

[11] Panizzolo FA, Galiana I, Asbeck AT, Siviy C, Schmidt K, Holt KG (2016). A biologically-inspired multi-joint soft exosuit that can reduce the energy cost of loaded walking. J Neuroeng Rehabil.

[12] Galle S, Malcolm P, Derave W, De Clercq D (2014). Enhancing performance during inclined loaded walking with a powered ankle-foot exoskeleton. Eur J Appl Physiol.

[13] Herr H (2009). Exoskeletons and orthoses: classification, design challenges and future directions. J. Neuroeng. Rehabil..

[14] Norris JA, Granata KP, Mitros MR, Byrne EM, Marsh AP (2007). Effect of augmented plantarflexion power on preferred walking speed and economy in young and older adults. Gait Posture.

[15] Jackson RW, Collins SH (2015). An experimental comparison of the relative benefits of work and torque assistance in ankle exoskeletons. J Appl Physiol.

[16] Sawicki GS, Ferris DP (2008). Mechanics and energetics of level walking with powered ankle exoskeletons. J Exp Biol.

[17] Koller JR, Jacobs DA, Ferris DP, Remy CD (2015). Learning to walk with an adaptive gain proportional myoelectric controller for a robotic ankle exoskeleton. J. Neuroeng. Rehabil..

[18] Wehner M, Quinlivan B, Aubin PM, Martinez-Villalpando E, Baumann M, Stirling L (2013). A lightweight soft exosuit for gait assistance.

[19] Galle S, Malcolm P, Collins SH, Speeckaert J, De Clercq D (2015). Optimizing robotic exoskeletons actuation based on human neuromechanics experiments: interaction of push-off timing and work.

[20] Ding Y, Panizzolo FA, Siviy CJ, Malcolm P, Galiana I, Holt KG (2016). Effect of timing of hip extension assistance during loaded walking with a soft exosuit. J Neuroeng Rehabil.

[21] Asbeck A, De Rossi SMM, Galiana I, Ding Y, Walsh C (2014). Stronger, smarter, softer: next-generation wearable robots. IEEE Robot Autom.

[22] Asbeck AT, De Rossi SMM, Holt KG, Walsh CJ (2015). A biologically inspired soft exosuit for walking assistance. Int J Robot Res.

[23] Margaria R (1968). Positive and negative work performances and their efficiencies in human locomotion. Int Zeitschrift fur Angew Physiol.

[24] Winter D (1983). Energy generation and absorption at the ankle and knee during fast, natural, and slow cadences. Clin Orthop Relat Res.

[25] Umberger BR (2010). Stance and swing phase costs in human walking. J R Soc Interface.

[26] Dick, T.J.M., Nuckols, R.W., Sawicki, G.S., 2017. Tuned or not? Ultrasound measurements of soleus fascicle dynamics during human walking with elastic ankle exoskeletons, in: American Society of Biomechanics.

[27] Huang T-WP, Kuo AD (2014). Mechanics and energetics of load carriage during human walking. J Exp Biol.

[28] Asbeck AT, Schmidt K, Galiana I, Wagner D, Walsh CJ (2015). Multi-joint Soft Exosuit for Gait Assistance.

[29] Lee S, Crea S, Malcolm P, Galiana I, Asbeck A, Walsh C (2016). Controlling negative and positive power at the ankle with a soft exosuit.

[30] Ding Y, Galiana I, Asbeck A, Quinlivan B, De Rossi SMM, Walsh CJ (2014). Multi-joint actuation platform for lower extremity soft exosuits. Int. Conf. Robot. Autom.

[31] Ding Y, Galiana I, Asbeck A, De Rossi S, Bae J, Santos T, et al. Biomechanical and Physiological Evaluation of Multi-joint Assistance with Soft Exosuits. TNSRE. 2016;25(2):119–30. doi:10.1109/TNSRE.2016.2523250, http://ieeexplore.ieee.org/abstract/document/7394183/. 10.1109/TNSRE.2016.2523250 26849868

[32] Asbeck AT, De Rossi SMM, Holt KG, Walsh CJ. A biologically inspired soft exosuit for walking assistance. Int J Rob Res. 2015;34(6):744–62. https://doi.org/10.1177/0278364914562476.

[33] Lee S, Crea S, Galiana I, Malcolm P, Walsh CJ (2016). Controlling negative and positive power at the ankle with a soft exosuit.

[34] Panizzolo F, Galiana I, Asbeck AT, Siviy C, Schmidt K, Holt KG, et al. A biologically-inspired multi-joint soft exosuit that can reduce the energy cost of loaded walking. J. Neuroeng. Rehabil.. Journal of NeuroEngineering and Rehabilitation; 2016 10.1186/s12984-016-0150-9 10.1186/s12984-016-0150-9 PMC4864923 27169361

[35] Malcolm P, Quesada RE, Caputo JM, Collins SH (2015). The influence of push-off timing in a robotic ankle-foot prosthesis on the energetics and mechanics of walking. J Neuroeng Rehabil.

[36] Galle S, Malcolm P, Derave W, De Clercq D (2013). Adaptation to walking with an exoskeleton that assists ankle extension. Gait Posture.

[37] Brockway JM (1987). Derivation of formulae used to calculate energy expenditure in man. Hum Nutr Clin Nutr.

[38] Cavagna GA (1975). Force platforms as ergometers. J Appl Physiol.

[39] Donelan JM, Kram R, Kuo AD (2002). Simultaneous positive and negative external mechanical work in human walking. J Biomech.

[40] Stanhope SJ, Kepple TM, McGuire DA, Roman NL (1990). Kinematic-based technique for event time determination during gait. Med Biol Eng Comput.

[41] Shamaei K, Member S, Member MC, Adams AA, Gregorczyk KN, Schiffman JM, et al. Effects of exoskeletal stiffness in parallel with the knee on the motion of the human body center of mass during walking. Seattle: ICRA; 2015. p. 5557–64.

[42] Voloshina AS, Ferris DP (2013). Biomechanics and energetics of running on uneven terrain. J Exp Biol.

[43] Hansen AH, Childress DS, Miff SC, Gard SA, Mesplay KP (2004). The human ankle during walking: implications for design of biomimetic ankle prostheses. J Biomech.

[44] Farris DJ, Sawicki GS. The mechanics and energetics of human walking and running: a joint level perspective. J R Soc Interface. 2011;9(66):110–8. https://doi.org/10.1098/rsif.2011.0182. 10.1098/rsif.2011.0182 PMC3223624 21613286

[45] Farris DJ, Sawicki GS (2012). Linking the mechanics and energetics of hopping with elastic ankle exoskeletons. J Appl Physiol.

[46] Jackson, R.W., Collins, S.H., 2015. An experimental comparison of the relative benefits of work and torque assistance in ankle exoskeletons, dataset http://biomechatronics.cit.cmu.edu/publications/Jackson_2015_JAP-%2D-Data.zip. 10.1152/japplphysiol.01133.2014 26159764

