# Supplementary figures and images for: Varying negative work assistance at the ankle with a soft exosuit during loaded walking
Source: J Neuroeng Rehabil. 2017 Jun 26;14:62. doi: 10.1186/s12984-017-0267-5 (PMC5485681; doi:10.1186/s12984-017-0267-5)

Exosuit hip/ankle  
force ratio

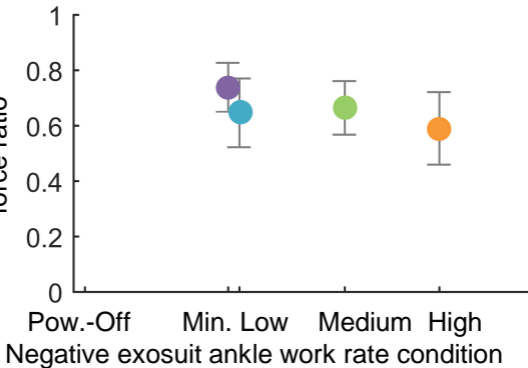

Supplement: Supplementary file 2 — Multi-articular strap hip versus ankle force ratio. Average force ratio from separate tests in three participants with an additional load cell on the proximal attachment of the multi-articular straps. Error bars are s.e.m. (PDF 36 kb) [file 12984_2017_267_MOESM2_ESM.pdf]

**A)**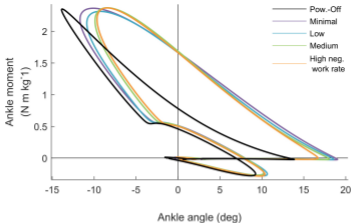**B)**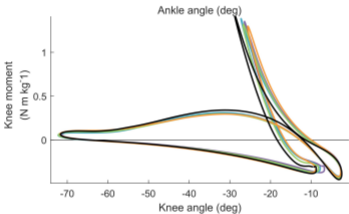**C)**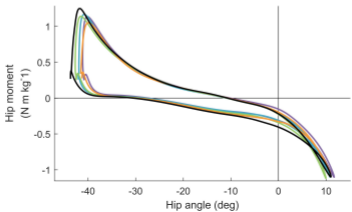

Supplement: Supplementary file 3 — Joint moments versus joint angle relationships. (A) Ankle joint moment versus ankle angle. (B) Knee moment versus knee angle. (C) Hip moment versus hip angle. Colored lines represent average time in conditions with different rates of negative work assistance. Black line is Powered-Off and shaded areas represent standard error. (PDF 2 kb) [file 12984_2017_267_MOESM3_ESM.pdf]

**A)**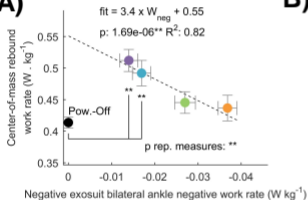**B)**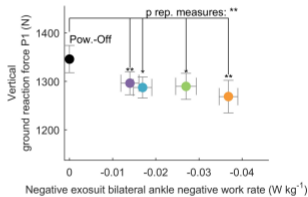

Supplement: Supplementary file 4 — Total body kinematics and kinetics timeseries. (A) Center-of-mass rebound work rate. (B) Vertical ground reaction force first peak. Dots are condition averages. Error bars are s.e.m. Dashed black line indicates linear fit from mixed-model ANOVA. Brackets indicate pairwise differences versus Powered-Off. Black dot represents Powered-Off reference condition. ** is p ≤ 0.01, * is p ≤ 0.05. (PDF 570 kb) [file 12984_2017_267_MOESM4_ESM.pdf]

**A)**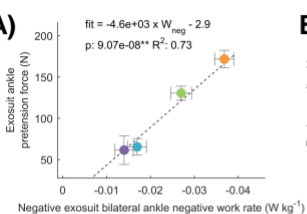**B)**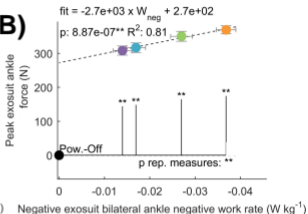**C)**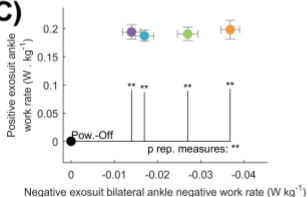

Supplement: Supplementary file 6 — Ankle actuation metrics. (A) Exosuit ankle pretension force. Exosuit ankle pretension force is defined as the peak force at the end of the negative work phase. (B) Peak exosuit ankle force. (C) Positive exosuit ankle work rate. Dots are condition averages. Error bars are s.e.m. Dashed black line indicates linear fit from mixed-model ANOVA. Brackets indicate pairwise differences versus Powered-Off. Black dot represents Powered-Off reference condition. ** is p ≤ 0.01, * is p ≤ 0.05. (PDF 870 kb) [file 12984_2017_267_MOESM6_ESM.pdf]

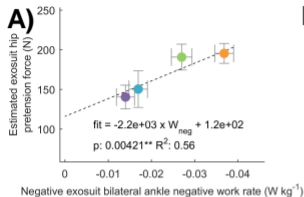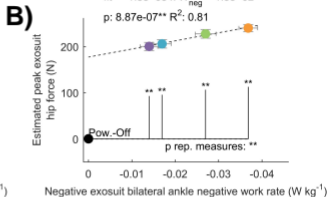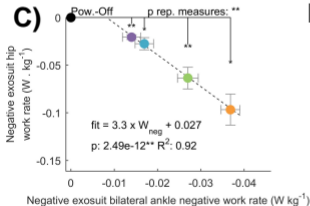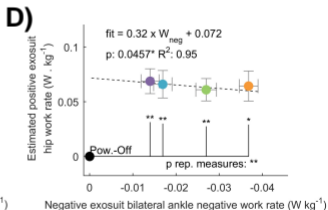

Supplement: Supplementary file 7 — Hip actuation metrics. (A) Exosuit hip pretension force. Exosuit hip pretension force is defined as the peak force at the end of the negative work phase. (B) Peak exosuit hip force. (C) Negative exosuit hip work rate. (D) Positive exosuit hip work rate. Dots are condition averages. Error bars are s.e.m. Dashed black line indicates linear fit from mixed-model ANOVA. Brackets indicate pairwise differences versus Powered-Off. Black dot represents Powered-Off reference condition. ** is p ≤ 0.01, * is p ≤ 0.05. (PDF 1 mb) [file 12984_2017_267_MOESM7_ESM.pdf]

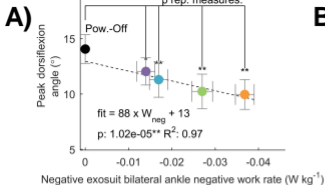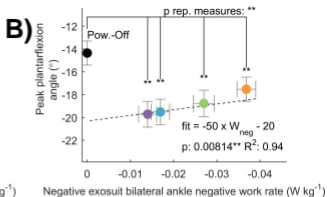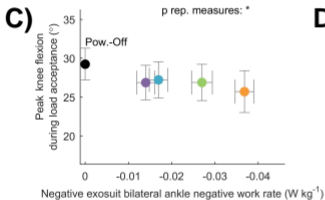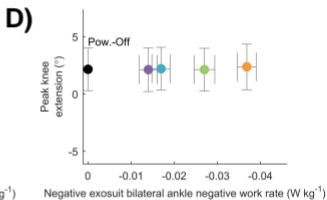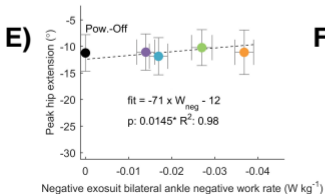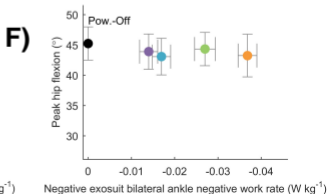

Supplement: Supplementary file 8 — Joint kinematics metrics. (A) Peak dorsiflexion. (B) Peak plantarflexion. (C) Maximum knee flexion during load acceptance. (D) Peak knee extension. (E) Peak hip extension. (F) Peak hip flexion. Dots are condition averages. Error bars are s.e.m. Dashed black line indicates linear fit from mixed-model ANOVA. Brackets indicate pairwise differences versus Powered-Off. Black dot represents Powered-Off reference condition. ** is p ≤ 0.01, * is p ≤ 0.05. (PDF 1 mb) [file 12984_2017_267_MOESM8_ESM.pdf]

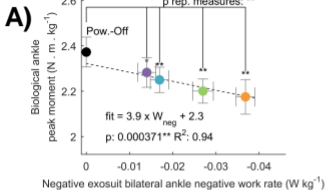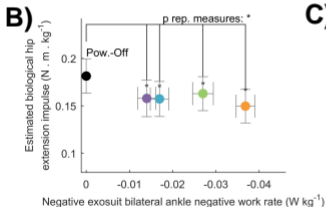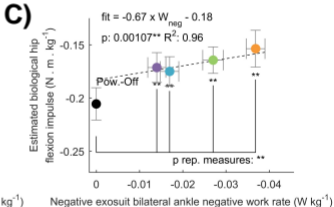

Supplement: Supplementary file 9 — Biological joint moment metrics. (A) Biological ankle peak plantarflexion moment. (B) Estimated biological hip extension impulse. (C) Estimated biological hip flexion impulse. Dots are condition averages. Error bars are s.e.m. Dashed black line indicates linear fit from mixed-model ANOVA. Brackets indicate pairwise differences versus Powered-Off. Black dot represents Powered-Off reference condition. ** is p ≤ 0.01, * is p ≤ 0.05. (PDF 891 kb) [file 12984_2017_267_MOESM9_ESM.pdf]

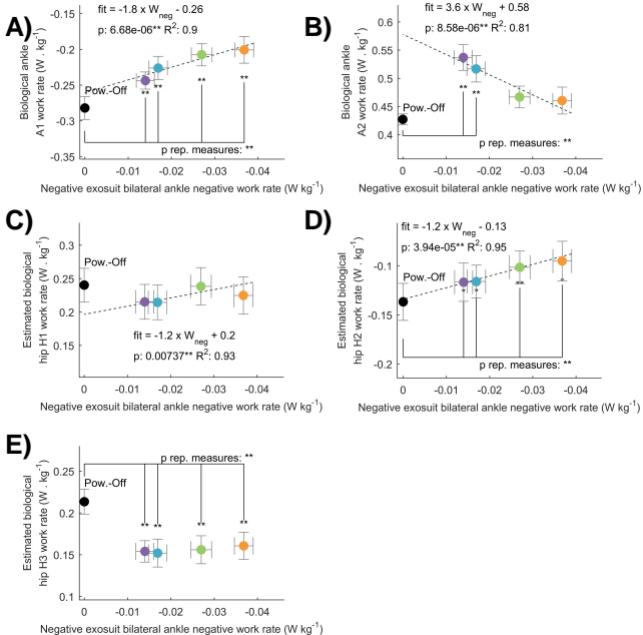

Supplement: Supplementary file 10 — Biological joint work metrics. (A) Biological ankle A1 work rate. (B) Biological ankle A2 work rate. (C) Estimated biological hip H1 work rate. (D) Estimated biological hip H2 work rate. (E) Estimated biological hip H3 work rate. Dots are condition averages. Error bars are s.e.m. Dashed black line indicates linear fit from mixed-model ANOVA. Brackets indicate pairwise differences versus Powered-Off. Black dot represents Powered-Off reference condition. ** is p ≤ 0.01, * is p ≤ 0.05. (PDF 1 mb) [file 12984_2017_267_MOESM10_ESM.pdf]
