# Supplementary material for: Varying negative work assistance at the ankle with a soft exosuit during loaded walking
Source: J Neuroeng Rehabil. 2017 Jun 26;14:62. doi: 10.1186/s12984-017-0267-5 (PMC5485681; doi:10.1186/s12984-017-0267-5)
Supplement: Supplementary file 5 — Exosuit weight split out by components. (PDF 13 kb) [file 12984_2017_267_MOESM5_ESM.pdf]

Segment	Weight			
2 boot attachments	136	g		
4 gyroscopes	92	g		
Subtotal for foot segments			228	g
2 calf wraps	188	g		
2 load cells	124	g		
4 ankle straps	84	g		
Bowden cable sections	80	g		
Subtotal for shank segments			476	g
1 waist belt	272	g		
4 hip straps	85	g		
Subtotal for waist segment			356	g
Total Mass			1060	g
